# Subclinical Hyperthyroidism Presenting as Urinary Frequency and Polyuria: A Case Report

**DOI:** 10.7759/cureus.88251

**Published:** 2025-07-18

**Authors:** Hameed H Salah, Elizabeth M Pines

**Affiliations:** 1 Anesthesiology, OhioHealth Doctors Hospital, Columbus, USA; 2 Internal Medicine, Edward Via College of Osteopathic Medicine, Blacksburg, USA; 3 Internal Medicine, Carilion Giles Community Hospital, Pearisburg, USA

**Keywords:** aspiration pneumonia, multinodular goiter, polyuria, subclinical hyperthyroidism, urinary frequency

## Abstract

Hyperthyroidism may present with a variety of symptoms including urinary frequency. This report discusses a case of subclinical hyperthyroidism presenting as generalized weakness, urinary frequency, polyuria, and intermittent diarrhea. However, this elderly patient had a complex past medical history including multinodular nontoxic thyroid goiter with tracheal deviation and multiple recent admissions for aspiration pneumonia, making the diagnosis of subclinical hyperthyroidism less obvious. The patient was managed with intravenous fluids and discharged on propranolol with recommendations to follow-up with endocrinology. This case demonstrates the importance of keeping hyperthyroidism on the differential for urinary frequency and polyuria without other clear etiologies.

## Introduction

Hyperthyroidism is a unique clinical entity with a plethora of possible presenting symptoms, including fatigue, tremor, weight loss, heat intolerance, sweating, and polydipsia [1]. Increased urinary frequency and polyuria are also possible presenting symptoms of hyperthyroidism, though far less frequent [2,3]. Research regarding the association between urinary frequency and hyperthyroidism was previously scarce, relying predominantly on case reports. One report found that a patient presenting with polyuria, both increased frequency and urinary volume, for over a month was found to have thyrotoxicosis [2]. Of note, there do not appear to be any case reports documenting subclinical hyperthyroidism presenting primarily as urinary frequency and polyuria to date.

However, there is a growing body of evidence to suggest hyperthyroidism directly causes increased urine production. Both hyper- and hypothyroidism have been associated with alterations in renal blood flow [4,5]. Multiple studies and case reports have found that patients with hyperthyroidism had a statistically significant higher estimated glomerular filtration rate (eGFR) and/or lower creatinine before initiating medical therapy compared to afterwards [6-8]. Other mechanistic pathways in which hyperthyroidism may cause polyuria include downregulation of aquaporin 1 and 2 [3]. 

It is not only overt hyperthyroidism that has this effect on renal function but subclinical hyperthyroidism as well. One study found both overt and subclinical hyperthyroidism had a decrease in eGFR after initiating therapy and that the change in eGFR was comparable [9]. Another study showed an inverse relationship between thyroid-stimulating hormone (TSH) and glomerular filtration rate (GFR). In the study of 398 kidney transplant recipients, elevated levels of TSH were associated with reduced GFR, even in euthyroid patients [10]. “For each increase of TSH by 1 µIU/mL, eGFR decreased by 1.34 mL/min [95% CI, −2.51 to −0.16; p = 0.03], corresponding to 2.2% eGFR decline” [10].

## Case presentation

An 82-year-old male presented to the ER complaining of severe generalized weakness, low blood pressure readings at home, cough, decreased appetite, intermittent diarrhea, and increased urinary frequency. He has a complex medical history including pneumoconiosis secondary to silica exposure, multinodular nontoxic thyroid goiter with tracheal deviation, chronic obstructive pulmonary disease (COPD), type 2 diabetes mellitus (T2DM) with peripheral neuropathy and long-term use of insulin, hypertension, hyperlipidemia, insomnia, transient ischemic attack (TIA), and recurrent hospitalizations in the last year for pneumonia. 

The patient has had recurrent pneumonia every three to seven months per report of the patient's family member and caretaker. He has been in and out of the hospital four times in the past month, and he was discharged from his last admission a week prior with prescriptions for a four-day course of Augmentin and doxycycline, as well as a tapering dose of steroids for pneumonia, all of which he was compliant with and completed. 

His multiple hospitalizations in the last year were for suspected aspiration pneumonia in the setting of the thyroid goiter with tracheal deviation. However, the patient has had multiple modified barium swallow studies performed without any signs of aspiration. Upon reviewing the records from his last few hospitalizations where he was said to have aspiration pneumonia, he did not have a new oxygen requirement, chest X-ray congruent with pneumonia, or abnormal pulmonary findings on physical exam. 

The patient’s family member reports that his symptoms this admission are the same as his last few admissions, with severe generalized weakness as the primary presenting symptom. Upon deeper historical investigation, the patient admitted to increased urinary frequency and urgency as well as passing excessive urine for over a month. The patient tried to stay hydrated at home by consuming various electrolyte drinks but reports it was difficult given his decreased appetite.

The patient’s home medications included aspirin 81 mg, budesonide-formoterol, ipratropium-albuterol, montelukast, famotidine, pantoprazole, sitagliptin-metformin, insulin degludec, dulaglutide, simvastatin, tramadol, quetiapine, citalopram, melatonin, and multi-vitamins. The patient denied any recent changes to his home medications and reported he his diabetic regimen has been stable for about a year. The last hemoglobin A1c available was 7.8, drawn about two weeks prior, showing relatively appropriate glucose control for his age.

Diagnostic workup

**Figure 1:**
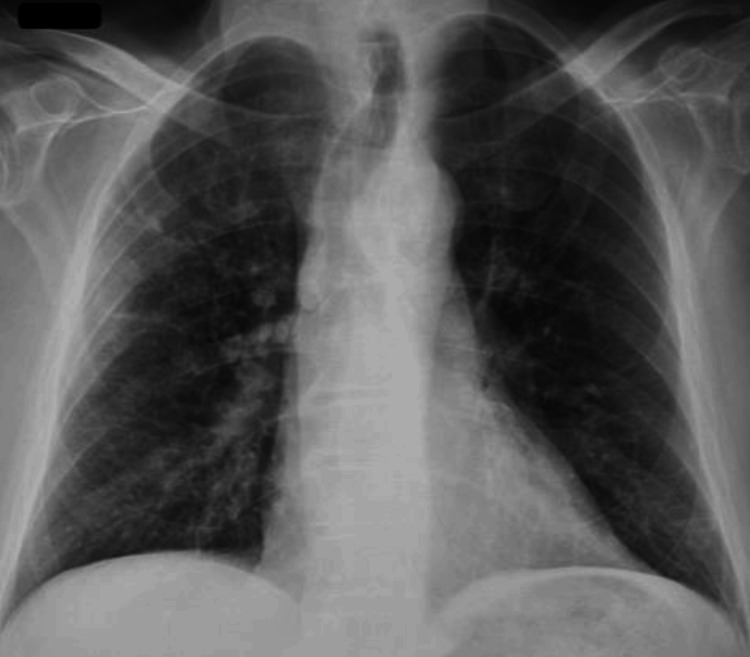
Chest X-Ray Official read: Diffuse interstitial opacities with suggestion of bibasilar ground-glass opacities. Finding may be seen with interstitial pneumonitis or mild pulmonary edema.

**Figure 2:**
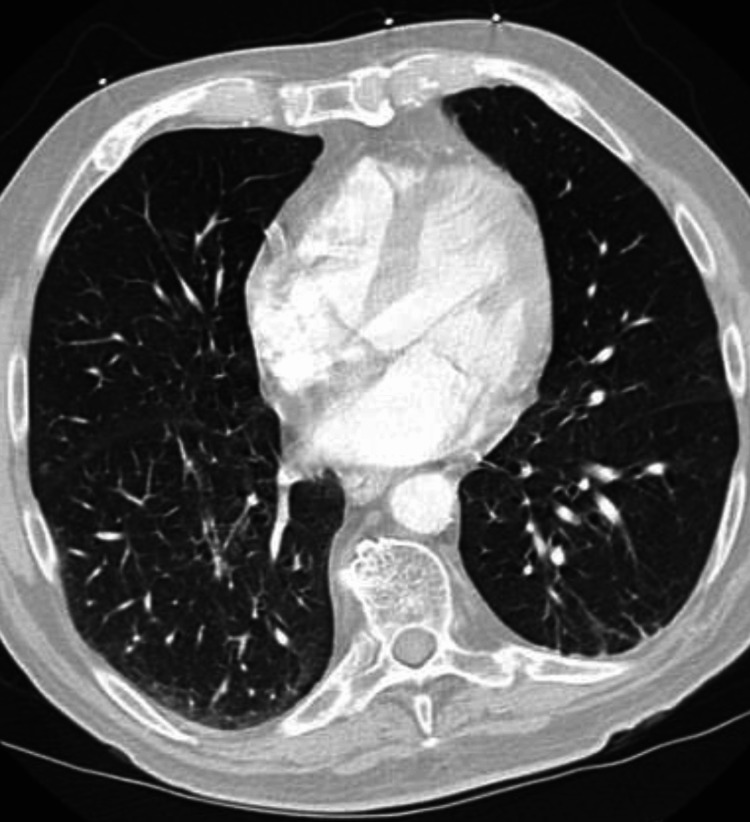
CT Angiogram of Chest Official read of lungs: Faint heterogeneous opacity of the right lung which is favored chronic.

**Figure 3:**
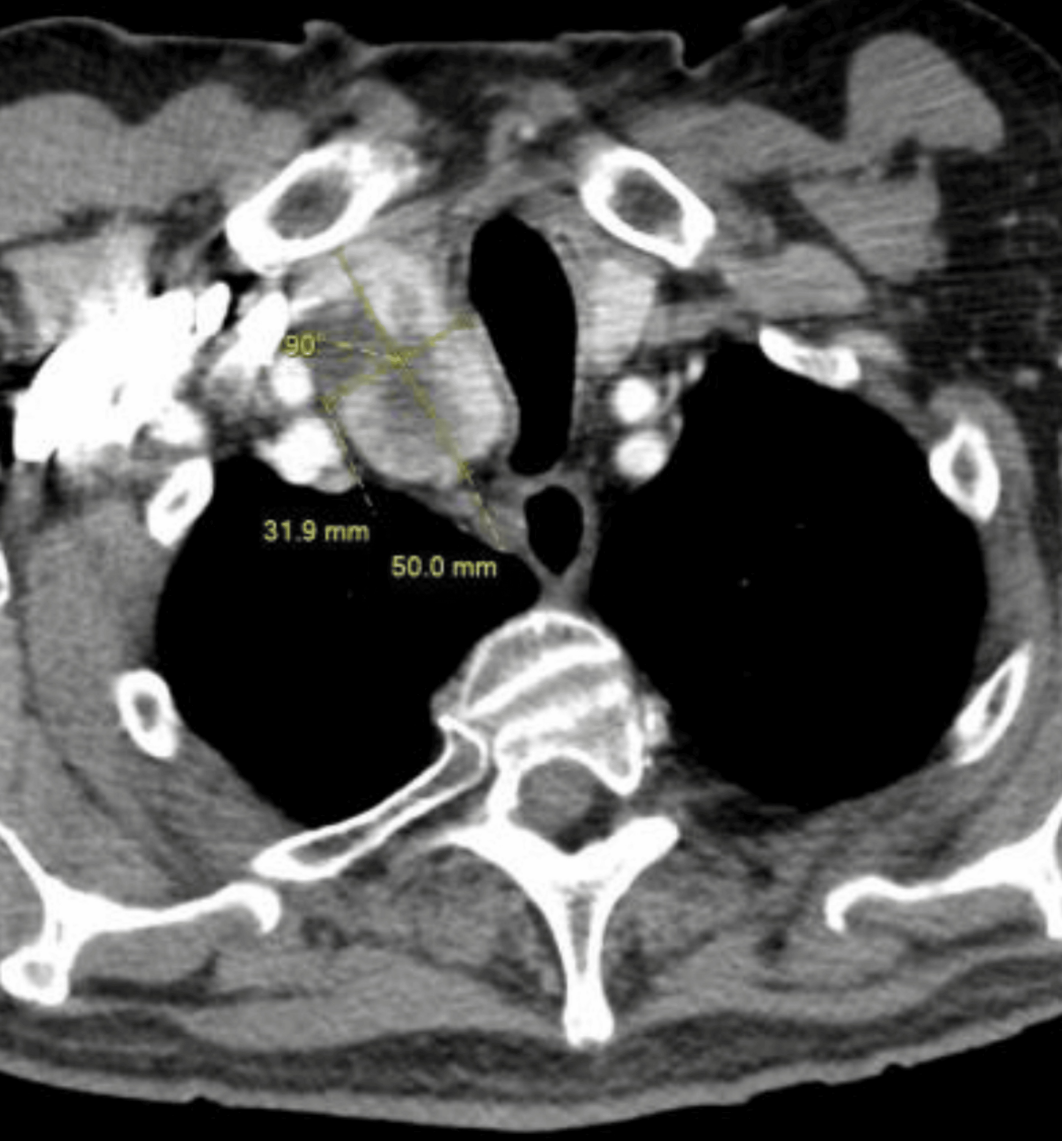
Thyroid goiter on CT chest Official read of thyroid: The right thyroid is severely enlarged and heterogeneous, mildly displacing the trachea leftward.

A chest X-ray and Computed Tomography (CT) scan were ordered to rule out pneumonia (Figures 1, 2, 3). The patient's radiographic findings appeared stable from prior admissions given his history of pneumoconiosis due to silica exposure. The patient’s pertinent laboratory findings are shown in Table 1. Vitals on admission included a blood pressure of 105/54 mmHg (mean arterial pressure of 71 mmHg), pulse of 68 beats per minute, respiratory rate of 14 breaths per minute, and an oxygen saturation of 95% on room air. Physical exam was non-contributory. Lungs were clear in all fields without crackles or wheezes. The patient was noted to have generalized weakness, without any isolated core muscle weakness. 

**Table 1:** Pertinent Laboratory Results

Laboratory Test	Institutional Reference Ranges	Day 0	Day 1	Day 2
Complete Blood Count (CBC)	-	-	-	-
White Blood Cell count	4.0-10.5 K/uL	14.7	11.8	-
Red Blood Cell count	4.5-5.3 M/uL	4.08	3.61	-
Hemoglobin	13-16 g/dL	10.5	9.3	-
Hematocrit	37%-49%	34.1	30.3	-
Mean Corpuscular Volume	78-98 fL	83.6	83.9	-
Mean Corpuscular Hemoglobin	27-34.6 pg	25.7	25.8	-
Mean Corpuscular Hemoglobin Concentration	33-37 g/dL	30.8	30.7	-
Mean Platelet Volume	9.4-12.4 fL	9.5	9.7	-
Red Cell Distribution Width	11.5%-14.5%	15.1	15	-
Platelets	130-400 K/uL	376	338	-
Basic Metabolic Panel (BMP)	-	-	-	-
Sodium	136-148 mmol/L	139	144	144
Potassium	3.5-5.2 mmol/L	5.6	4.7	5.1
Bicarbonate	20-32 mmol/L	26	30	30
Urea Nitrogen	7-23 mg/dL	26	22	18
Creatinine	0.55-1.02 mg/dL	1.13	1.04	0.96
Albumin	3.2-5.0 g/dL	3.3	-	-
Glucose, serum	74-106 mg/dL	205	97	87
Bun/Creatinine	7-20	23	21.2	18.8
Glomerular Filtration Rate, Estimated	>60 mL/min/1.73 m^2	63	70	81
Iron Panel	-	-	-	-
Iron	65-175 ug/dL	-	20	-
Ferritin	30-400 ng/mL	-	29.6	-
Total Iron-Binding Capacity	250-425 ug/dL	-	218	-
Unsaturated Iron-Binding Capacity	153-344 ug/dL	-	198	-
Transferrin Saturation	9.2%-49.1%	-	9	-
Folate	>5.38 ng/mL	-	15.52	-
Vitamin B-12	211-911 pg/mL	-	616	-
Other	-	-	-	-
Troponin High Sensitivity	<57 ng/L	<4	-	-
Pro–B-Type Natriuretic peptide	<450 pg/mL	141	-	-
Hemoglobin A1c	<5.7%	6.5	-	-
Thyroid Stimulating Hormone	0.55-4.78 uIU/mL	-	0.25	-
Free thyroxine	0.8-1.8 ng/dL	-	-	1.5
Free T3	2.3-4.2 pg/mL	-	-	2.6
Reverse T3	8-25 ng/dL	-	-	34

A TSH was checked and found to be low at 0.25. Further testing was ordered, though the results only returned after discharge, including a free T3 of 2.6, an elevated reverse T3 of 34, and a free T4 of 1.5. These results together suggest that the patient had subclinical hyperthyroidism. 

Other notable findings include decreased hemoglobin, iron, and transferrin saturation, increased potassium, and increased creatinine to 1.13 from a baseline of 0.85 seen on prior records. A urinalysis performed was unremarkable and later a urine culture returned without any growth. Urinary tract infection as the cause of the patient’s urinary frequency was ruled out. 

Management

The patient was admitted to the hospital for generalized weakness and possible aspiration pneumonia, which was later ruled out by CT. During the admission, additional diagnoses of acute kidney injury (AKI), iron deficiency anemia, hyperkalemia, and orthostatic hypotension were made. 

As only the TSH had returned during the admission, the patient was discharged on propranolol for suspected hyperthyroidism. The remainder of the thyroid panel later revealed the patient had subclinical hyperthyroidism. The patient was advised to follow up with endocrinology for further management. 

Intravenous (IV) fluids effectively resolved the patient's generalized weakness, AKI, hyperkalemia, and improved the orthostatic hypotension. Iron deficiency anemia was managed with intravenous iron sucrose and oral supplementation initiated upon discharge.

## Discussion

As noted previously, there is a linear relationship between TSH levels and eGFR; low TSH is associated with increased GFR [10]. Even subclinical hyperthyroidism has been associated with alterations in eGFR comparable to those seen in overt hyperthyroidism [9]. We suspect that this patient experienced increased urinary frequency, polyuria, and intermittent diarrhea because of his subclinical hyperthyroid state. Other reports have described similar presentations, including excessive urination for over a month in patients found to have hyperthyroidism [2]. With the increased urine production and inadequate hydration at home, this patient likely became hypovolemic and exhibited orthostatic hypotension, generalized weakness, and developed AKI. With IV fluid hydration, as well as the use of propranolol, his volume status normalized, and his generalized weakness resolved.

There is a possibility that some of the patient’s most recent hospitalizations for aspiration pneumonia were due to his subclinical hyperthyroidism. As mentioned previously, several admissions had radiographic, barium swallow, and physical exam findings that were incongruent with aspiration pneumonia. However, the thyroid mass with tracheal deviation is an infrequent co-morbidity and may have led to an anchoring bias and bandwagon effect. Each readmission for aspiration pneumonia encouraged the assumption that the next admission would be for the same reason. With each admission, the patient received antibiotics and fluids, resulting in clinical improvement of his symptoms. 

Polyuria secondary to uncontrolled diabetes was considered. However, given his relatively well-controlled A1c, lack of new diabetic medications or changes to his diabetic regimen, including glucagon-like peptide-1 (GLP-1) use, and absence of prior admissions in the past year noting significant hyperglycemia, this cause of polyuria was ruled out.

This case report is limited in a number of ways. Unfortunately, the patient was lost to follow-up at the time of first drafting this manuscript and access to the medical record is no longer available to the authors at the time of peer review. It is unclear if the patient had long-term clinical improvement in symptoms after initiating propranolol therapy. It is unknown if the patient was evaluated by endocrinology to determine if his multinodular goiter had undergone toxic transformation, leading to his subclinical hyperthyroid state, a phenomenon documented in the literature [11].

## Conclusions

In this case report, we discuss an atypical presentation of subclinical hyperthyroidism presenting as urinary frequency and polyuria, a rare clinical presentation. The diagnosis is easily missed, especially in patients with complex medical histories. In our patient, a history of frequent hospitalizations for aspiration pneumonia in the setting of a thyroid mass with tracheal deviation made the diagnosis of subclinical hyperthyroidism less clear. However, given that this patient’s physical exam and radiographic findings, as well as multiple barium swallow studies, were incongruent with aspiration pneumonia, an alternative diagnosis was sought and found. This case report is limited as no follow-up information is available or whether long-term clinical improvement was achieved with propranolol therapy. We aim to emphasize the importance of keeping hyperthyroidism on the differential diagnosis in the setting of urinary frequency without other clear etiologies. 
